# A pilot study showing differences in glycosylation patterns of IgG subclasses induced by pneumococcal, meningococcal, and two types of influenza vaccines

**DOI:** 10.1002/iid3.22

**Published:** 2014-05-22

**Authors:** Anne Cathrine Vestrheim, Anders Moen, Wolfgang Egge-Jacobsen, Leon Reubsaet, Trine Grønhaug Halvorsen, Diane Bryant Bratlie, Berit Smestad Paulsen, Terje Einar Michaelsen

**Affiliations:** 1 Department of Bacteriology & Immunology, Norwegian Institute of Public HealthOslo, Norway; 2 Department of Pharmaceutical Chemistry, School of Pharmacy, University of OsloOslo, Norway; 3 Department of Molecular Biosciences, University of OsloOslo, Norway

**Keywords:** Antibodies, human and glycosylation, immunology, vaccination

## Abstract

The presence of a carbohydrate moiety on asparagine 297 in the Fc part of an IgG molecule is essential for its effector functions and thus influences its vaccine protective effect. Detailed structural carbohydrate analysis of vaccine induced IgGs is therefore of interest as this knowledge can prove valuable in vaccine research and design and when optimizing vaccine schedules. In order to better understand and exploit the protective potential of IgG antibodies, we carried out a pilot study; collecting serum or plasma from volunteers receiving different vaccines and determining the IgG subclass glycosylation patterns against specific vaccine antigens at different time points using LC-ESI-MS analysis. The four vaccines included a pneumococcal capsule polysaccharide vaccine, a meningococcal outer membrane vesicle vaccine, a seasonal influenza vaccine, and a pandemic influenza vaccine. The number of volunteers was limited, but the results following immunization indicated that the IgG subclass which dominated the response showed increased galactose and the level of sialic acid increased with time for most vaccinees. Fucose levels increased for some vaccinees but in general stayed relatively unaltered. The total background IgG glycosylation analyzed in parallel varied little with time and hence the changes seen were likely to be caused by vaccination. The presence of an adjuvant in the pandemic influenza vaccine seemed to produce simpler and less varied glycoforms compared to the adjuvant-free seasonal influenza vaccine. This pilot study demonstrates that detailed IgG glycosylation pattern analysis might be a necessary step in addition to biological testing for optimizing vaccine development and strategies.

## Introduction

A robust IgG response is generally a hallmark of an effective vaccine against invasive pathogens [1–3]. IgG molecules contain an N-linked oligosaccharide moiety bound to asparagine 297 on each chain in the Fc part of the molecule and several studies have demonstrated that the presence of carbohydrates in the Fc region has direct influence on effector functions and thus the vaccine protective effect of IgG. Remarkably, IgG can have a dramatic anti-inflammatory effect, shown by the ability of intravenous immunoglobulin G; IVIg (pooled immunoglobulin from healthy donors) to treat auto-immune diseases and chronic inflammatory diseases [4]. The presence of sialic acid at the Fc glycosylation site seems necessary for the anti-inflammatory effect of IVIgs [5]. Galactose levels change during the life cycle and during disease states, such as Chrohn's disease or rheumatoid arthritis [6]. Strikingly, women suffering from rheumatoid arthritis often display increased galactose-rich IgG during pregnancy combined with a remission in arthritis symptoms [7]. Galactose-rich mouse IgG1 elicits anti-inflammatory activity by engaging the inhibitory FcγRIIb, further elucidating the glycan importance for IgG function [8]. Fucose appears to have an essential role in antibody dependent cellular cytotoxicity (ADCC) and opsonophagocytosis. Monoclonal humanized IgG1 with low levels of core-fucose shows dramatic higher ADCC and opsonophagocytosis activity compared to the corresponding fucose-rich antibodies due to an increased affinity to FcγRIIIa and FcγRIIIb, respectively [9–12]. The levels of bisecting GlcNAc (*N*-acetylglucosamine) in human IgG do not generally exceed 10% of total IgG, but when reaching 48–71%, increased ADCC activity was observed in a chimeric mouse/human α-CD20 IgG1 antibody [13–15]. The significance of IgG glycosylation is therefore evident, so we asked the question how are these glycosylation patterns affected, if at all, following vaccination?

Immunization studies have been carried out in mice, where mice immunized with bovine serum albumin (BSA) displayed anti-BSA titers with reduced levels of galactose and mice immunized with ovalbumin showed increased fucosylation for each booster dose [16,17]. The glycosylation patterns of IgG1 following immunization against influenza and tetanus in humans have recently been published, and showed that antigen-specific IgG1 had increased levels of sialic acid and galactose following immunization [18]. Apart from this, we have found no other studies investigating IgG glycosylation patterns in humans following immunization and would like to that area with this study.

Different vaccine antigens have different pharmaceutical formulations to combat different types of pathogens. Meningococcal outer membrane vaccines mainly induce an IgG1 and IgG3 response [19], while polysaccharide vaccines, like pneumococcal vaccines, mainly produce an IgG2 response [20]. The main IgG subclass produced by influenza vaccines is likely to be IgG1. Different IgG subclass responses may represent different glycosylation patterns, and if these were to change over the time course of vaccination that could greatly influence the vaccine effect. Detailed analysis of glycosylation changes could therefore contribute to a more comprehensive understanding of vaccine immune protection and this could provide valuable information for vaccine research, development, and design, and finally be used in the foundation upon which recommended vaccine schedules are formed.

Mass spectrometry is often used to determine the glycosylation pattern of IgG from serum or plasma samples [21–23]. The glycan moiety is covalently linked at asparagine 297 on the Fc region of the antibody [7] and the carbohydrates fucose, sialic acid, and galactose are of particular interest. In our study, serum IgG glycosylation patterns of all relevant IgG subclasses from four different vaccines were analyzed, a pneumococcal capsule polysaccharide vaccine, a meningococcal outer membrane vesicle vaccine, a seasonal influenza vaccine and a pandemic influenza vaccine. By choosing four different formulations with various antigens, changes in the glycosylation patterns as a consequence of formulation or antigen type could be revealed. Blood samples were taken at different time points following vaccination and specific antibodies and total background IgG were analyzed and the glycosylation patterns determined. Our method permitted the differentiation of all the IgG subclasses of interest. The number of volunteers receiving each vaccine was limited, but the results could provide indications of glycosylation pattern differences worth pursuing in larger studies.

## Materials and Methods

### Sample collection

The IgG responses following immunization against pneumococci, meningococci and influenza (seasonal and pandemic) were studied. For the pneumococcal vaccine and both influenza vaccines, the volunteers involved in the project received these vaccinations as part of their regular vaccination regimes and kindly donated their blood. The plasma and serum from the meningococcal vaccine volunteers were remnants from a previous national study that was approved by the Norwegian Medicines Control Authority and the Regional Ethical Committee for Medical Sciences, South East Norway. A new application for use of these samples in our study was approved by the Regional Ethical Committee for Medical Sciences, South East Norway. The meningococcal vaccine was given as three or four doses, while the other vaccines were given as one dose only. The pneumococcal vaccine was a 23-valent pneumococcal polysaccharide vaccine; Pneumovax® (Sanofi Pasteur MSD, Brussels, Belgium) and the meningococcal vaccine was an outer membrane vesicle vaccine (88% protein, 5% LPS, and 7% phospholipids) with Al(OH)_3_ as adjuvant against group B meningococci (MenBVac®) [24–26]. Outer membrane vesicles (OMV) are spherical lipid bilayer vesicles isolated from the outer membrane of Gram-negative bacteria by detergent treatment and centrifugation [19,27,28]. The influenza vaccines were split, hemagglutinin based vaccines; the pandemic influenza vaccine, Pandemrix® (GlaxoSmithKline Biologicals S.A., Rixensart, Belgium) was based on strain A/California/7/2009 [29]. Different seasonal influenza vaccines were used, but they were all inactivated influenza virus for 2009/2010, 2010/2011, and 2011/2012, comprising Vaxigrip® (Sanofi Pasteur MSD), Begrivac® (Novartis Vaccines and Diagnostics GmbH & Co. KG, Marburg, Germany), and Influvac® (Abbott Biological B.V., Olst, Holland). The main differences between the pandemic and seasonal influenza vaccines were the dose (3.75 µg HA for the pandemic and 15 µg HA for the seasonal) and the presence of an adjuvant in the pandemic vaccine only.

The number of volunteers varied between the different vaccines; seven meningococcal vaccinees, four pneumococcal vaccinees, and five pandemic influenza vaccinees. There were three seasonal influenza vaccine volunteers. One volunteer received the vaccine for one season only, one volunteer received the vaccine for 2 consecutive years and the third volunteer received the vaccine for three consecutive years. Blood samples were collected each year, resulting in a total of six sets of seasonal influenza glycosylation results. For the pneumococcal and both types of influenza vaccines, three general time points were chosen for blood collection, day 0; hours before the first vaccination, and day 30 and day 90 post-vaccination. Deviations from this did occur to accommodate the schedules of the vaccinees. Subclass quantitation confirmed that IgG2 was the main subclass response for the pneumococcal vaccine and IgG1 and IgG3 for the other vaccines (see Supplementary Table S1 shows meningococcal results only). It was not possible to obtain serum from day 90 from two of the pneumococcal vaccinees; hence day 0, day 14, and day 26 were analyzed in this case. As the results were quite similar between day 14, day 26, and day 112 for the other vaccinees in this group, the time points for those two vaccinees were still labeled day 30 and day 90 for ease of presentation. For some vaccines, additional time points were collected. For the five pandemic influenza vaccinees, three were aged 26–30 (vaccinees # 005, # 006, and # 007) and two were aged 65+ (vaccinees # 008 and # 009). These numbers were too small for statistical analysis, but indications of trends could be seen.

For the meningococcal vaccinees, plasma samples from seven vaccinees were analyzed from three different time points; visit 4, 6, and 7 (see Table1). For vaccinee # 022, five additional time points were analyzed; eight visits in total (see Table1).

**Table 1:** The vaccine schedule for the meningoccocal vaccine. Doses were given at visit 1 (first dose), visit 2 (second dose), visit 4 (third dose), and visit 6 (fourth dose)

Visit number	Time point after vaccination	Type of sample
Visit 1	Day 0–first dose	Serum
Visit 2	6–8 weeks after the first dose	Serum
Visit 3	6–8 weeks after the second dose	Serum
Visit 4	1–2 weeks after the third dose	Plasma
Visit 5	6–8 weeks after the third dose	Serum
Visit 6	10–12 months after the third dose	Plasma
Visit 7	1–2 weeks after the fourth dose	Plasma
Visit 8	4–6 weeks after the fourth dose	Serum

**Figure 1:**
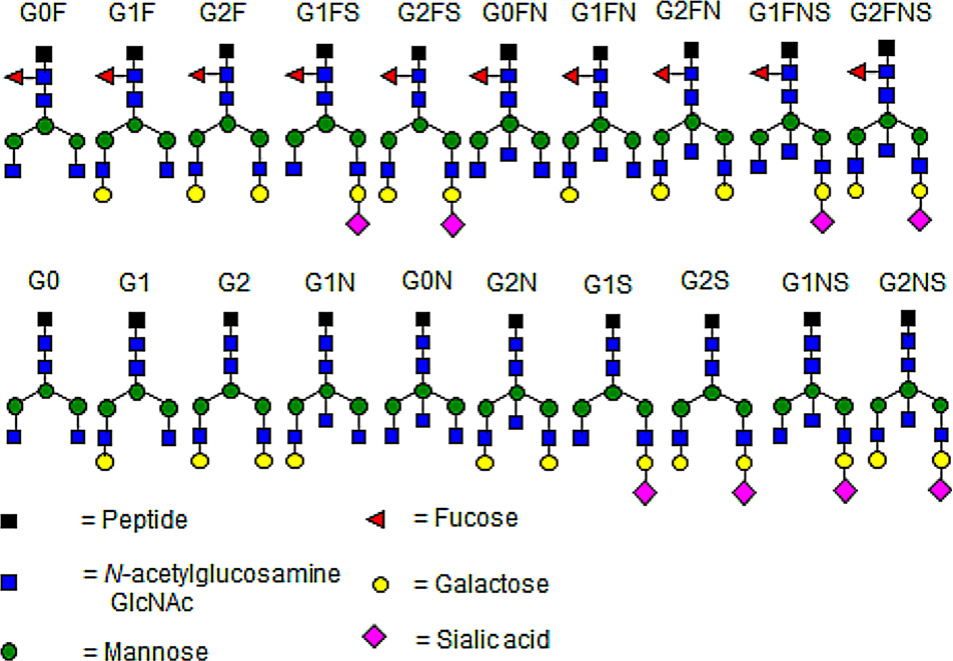
The 20 different glycoforms studied in this report. Black square = peptide, blue square = *N*-acetylglucosamine (GlcNAc), green (dark gray) circle = mannose, red triangle = fucose, yellow (light gray) circle = galactose, and pink diamond = sialic acid.

**Figure 2:**
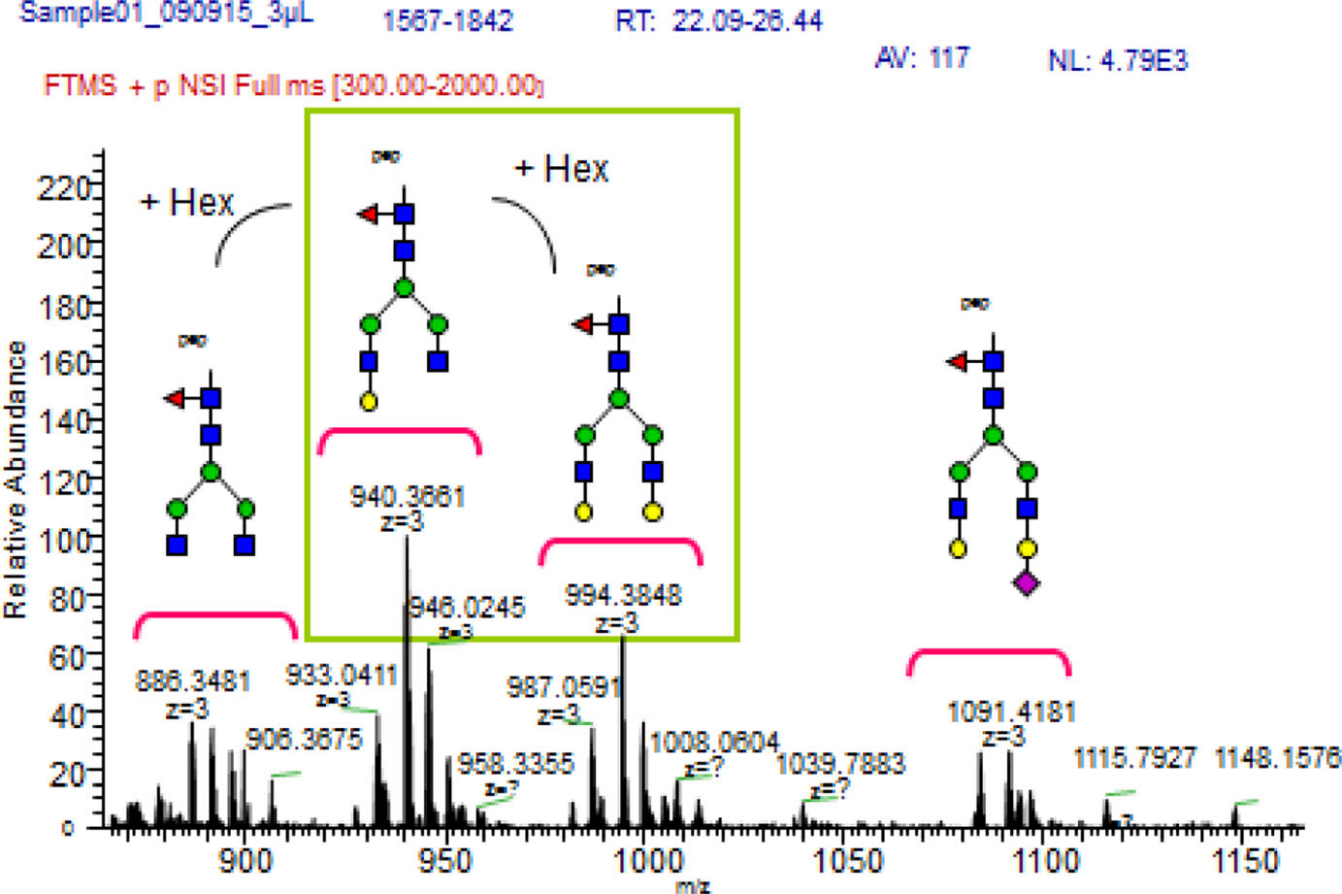
MS glycanspecter of an IgG3 molecule showing the relative abundance of the most common glycoforms. Starting from the left, the glycoforms shown are G0F, G1F, G2F, and finally G2FS. Black square = peptide, blue square = *N*-acetylglucosamine (GlcNAc), green (dark gray) circle = mannose, red triangle = fucose, yellow (light gray) circle = galactose, and pink diamond = sialic acid.

**Figure 3:**
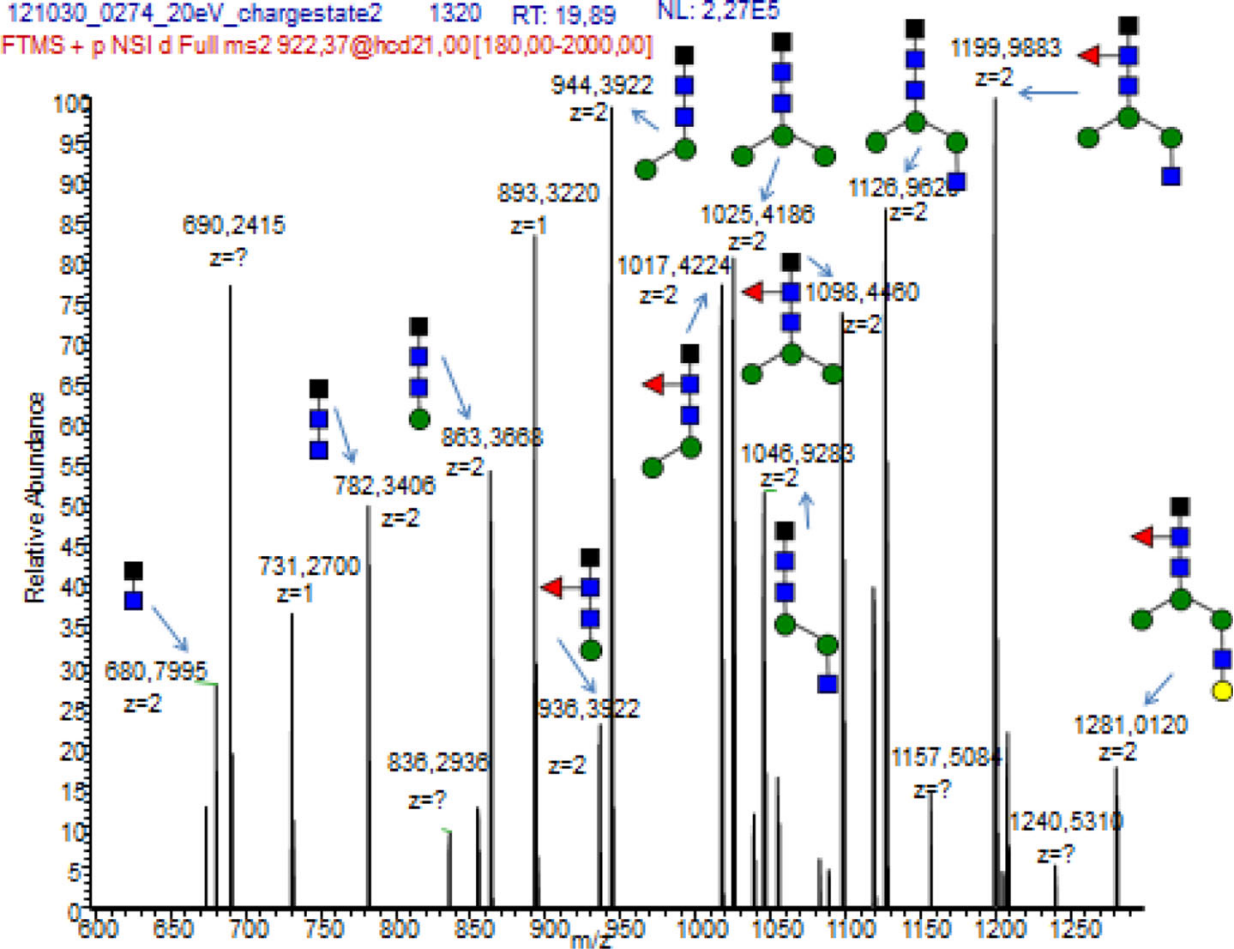
MS/MS glycanspecter of an IgG3 molecule where the peaks for the different carbohydrate compositions are shown. MS/MS or tandem mass spectrometry fragments the ions so the structure and composition of the molecule can be determined. To the far right, the peak corresponds to a carbohydrate composition of a peptide, three GlcNAc's, three mannose's, one fucose, and one sialic acid. The peak to the left of that corresponds to the same molecule, but it has been fragmented further and is without the sialic acid. Fucose has been removed in the peak to the left of that, and the fragmentation continues in this manner. Using MS/MS, the structure of the molecule can be determined. Black square = peptide, blue square = *N*-acetylglucosamine (GlcNAc), green (dark gray) circle = mannose, red triangle = fucose, yellow (light gray) circle = galactose, and pink diamond = sialic acid.

The samples studied in this report were all analyzed using LC-ESI-MS, looking for 20 different glycoforms (see Fig. 1) [21,30]. Glycoforms present with charge states dependent on the number of protons, and two charges states were found for this study, charge state 2 [M+2H]^2+^ with two protons and charge state 3 [M+3H]^3+^ with three protons, giving two options for each IgG1 or IgG3 glycoform (see Table2). The MS specter provides a picture of the relevant abundance of the different glycoforms, as shown for four IgG3 glycoforms (see Fig. 2). The peaks were integrated and the values for each glycoform in charge state 2 and 3 were combined, and the values for all glycoforms added together, and from this the percentage distribution of each glycoform was calculated. MS/MS (MS^2^) was carried out to confirm that the mass belonged to the correct glycoform (see Fig. 3). Glycoforms that represented less than 1% of the total distribution were excluded from the graphs. The background IgG was also analyzed. It proved difficult to differentiate between IgG2 and IgG3 in mass spectrometry, as for all but one vaccine; the amino acid sequence in the peptide generated by trypsin was identical; EEQFNSTFR. One meningococcal vaccinee had IgG3 antibodies with a different amino acid sequence, EEQYNSTFR. The mass to charge values for that IgG3 type can be found in Supplementary Table S2. The vaccine serum was separated to include an IgG1/IgG2/IgG4 fraction by elution from the protein A column and an IgG3 fraction eluted from the protein G column.

**Table 2:** Displaying mass to charge (*m*/*z*) of 20 IgG1, IgG2, and IgG3 glycoforms

	IgG1		IgG2 and IgG3	
		
	Chargestate 2	Chargestate 3	Chargestate 2	Chargestate 3
				
	IgG1 EEQYNSTYR		IgG2 and IgG3 EEQFNSTFR	
G0F	1317.5270	878.6891	1301.5320	868.0235
G1F	1398.5530	932.7044	1382.5581	922.0411
G2F	1479.5794	986.7220	1463.5848	976.0587
G1FS	1544.1007	1029.7362	1528.1058	1019.0729
G2FS	1625.1271	1083.7538	1609.1322	1073.0905
G0FN	1419.0663	946.3799	1403.0714	935.7166
G1FN	1500.0927	1000.3975	1484.0978	989.7342
G2FN	1581.1191	1054.4151	1565.1242	1043.7518
G1FNS	1645.6404	1097.4293	1629.6455	1086.7660
G2FNS	1726.6668	1151.4469	1710.6719	1140.7836
G0	1244.4976	830.0008	1228.5027	819.3375
G1	1325.5240	884.0184	1309.5291	873.3551
G2	1406.5504	938.0360	1390.5555	927.3727
G1N	1427.0637	951.7115	1411.0688	941.0483
G0N	1346.0373	897.6939	1330.0420	887.0307
G2N	1508.0901	1005.7291	1492.0952	995.0659
G1S	1471.0717	981.0502	1455.0768	970.3869
G2S	1552.0981	1035.0678	1536.1032	1024.4045
G1NS	1572.6114	1048.7433	1556.6165	1038.08
G2NS	1653.6378	1102.7609	1637.6430	1092.098

### IgG subclass determination

A method to quantitate the subclasses of the meningococcal vaccine response has previously been described [19]. Briefly, serum or plasma samples and control standards (specific IgG1 and IgG3 antibodies against OMV) were applied to OMV-coated microtiter plates and incubated. Subclass specific monoclonal antibodies were obtained from the WHO/International Union of Immunological Societies (IUIS) Immunoglobulin Subcommittee, IgG1 (HP 6012), IgG2 (HP 6002), and IgG3 (HP 6050) [19]. The plates were read at 405 nm using a Precision microplate reader from Molecular Devices. The same principle was applied to detect the influenza and pneumococcal IgG subclass vaccine response, but as no subclass specific antibodies were available, only the amount of one IgG subclass compared to another could be determined.

### Antibody capture

Microtiter plates were coated with 100 µL antigen in PBS and left for 48 h at 4°C. For the meningococcal vaccine serum, 4 µg/mL OMV antigen made from the Norwegian strain 44/76 was used. For the other vaccines, 1 µg/mL of Pneumovax® pneumococcal capsule polysaccharide vaccine (Sanofi Pasteur MSD), 1.5 µg/mL of Begrivac® seasonal influenza vaccine (Novartis) and 2 µg/mL of Pandemrix® pandemic influenza vaccine (Glaxo-SmithKline) were used. The plates were washed with 300 µL distilled water × 5. The plates were vigorously knocked against a bench covered in cell paper to make sure unbound liquid was removed after each wash cycle. Three hundred microliters of blocking buffer (1% dried milk in PBS/azid) was added to each well, followed by a 1 h incubation at 37°C and a wash with 300 µL distilled water × 5. The plasma samples were diluted 1 in 2.5 and the serum samples 1 in 5 with 5 M NaCl (plasma samples were previously diluted 1:1 during the isolation process) before they were added to the wells and incubated for 2 h at 37°C to allow antigen–antibody binding. The plates were washed with 300 µL 1 M NaCl × 3 and 300 µL distilled water × 2, sealed with tape and refrigerated pending trypsin digestion.

### Protein A and Protein G columns were used to isolate background total IgG

One milliliter of HiTrap protein A and 1 mL HiTrap protein G columns from GE Healthcare were coupled together with protein A as the first application column. By this procedure; IgG1, IgG2, and IgG4 bound to the protein A column while IgG3 passed through and bound to the protein G column. ELISA quantitation confirmed a successful isolation as only negligible traces of IgG3 were found in the eluates from the protein A column (data not shown).

Three hundred seventy-five microliters of serum mixed with 625 µL of PBS/azid were added to the columns and left at room temperature for 10 min. One milliliter of PBS/azid was then injected and incubated at room temperature for 10 min. Ten milliliter of PBS/azid was then washed through the columns to remove any unbound proteins.

The two columns were separated and flushed with an additional 5 mL PBS/azid before elution. IgG3 was eluted with 0.1 M glycin/HCl (pH 2.8) from the protein G column while IgG1, IgG2, and IgG4 were eluted from the protein A column with 0.2 M NaAc (pH 4.0). In total, 5 mL of elution buffer was used for each column, giving five vials with 1 mL eluate in each, neutralized with 1.5 M TRIS pH 8.5.

### Trypsin digestion before MS analysis

The background IgG samples were digested with trypsin in Nanosep® Centrifugal Devices (10K, blue, OD010C33, Pall Corporation, New York, USA). Approximately, 50 µg of antibodies were transferred to Nanosep devices. The devices were centrifuged for 5 min at 13,000*g* at 4°C and the bottom contents were discarded. One hundred twenty microliters of Trypsin digestion buffer (50 mM ammonium bicarbonate with 15% acetonitrile) was added to each well or Nanosep® device, incubated for 5 min at 80°C and sonicated in a water bath for 30 sec. Six hundred nanograms of trypsin was added to each well and 1 µg trypsin was added to each centrifugal device (sequencing grade modified trypsin from Promega (Madison, Wisconsin, USA) (V5111) or Roche Diagnostics (Basel, Switzerland) (11418025001)) and sonicated for 30 sec in a water bath, followed by an overnight incubation at 37°C. The following day the centrifugal devices were centrifuged at 13,000*g* at 4°C for 5 min and the separation filters removed. The content of the wells were transferred to Eppendorf tubes (Rainin LiteTouch™ 1.7 mL Microcentrifuge Tube LTT-170_B (17011862)) and the extracts were dried using a Speedvac centrifuge (Maxi dry Iyo F.D.1.0, Heto-Holten, Allerød, Denmark) for approximately 2 h. Seventeen microliters of 0.1% formic acid was added to each Eppendorf tube and quickly centrifuged, followed by 30 sec sonication and finally centrifuged at 16,000*g* for 10 min at 4°C. Fifteen microliters was then transferred to MS vials (VWR International microvials PP 0.3 mL with short thread, Cat. No. 548-0440 and VWR International Screw cap PP transparent, 9 mm silic. white/PTFE r, Cat. No. 548-0034) and the vials were consequently stored at −20°C until analysis.

### Mass spectrometry analysis of trypsin digested IgG samples

The samples were analyzed using LC-ESI Orbitrap and LC separation was carried out using Agilent 1200 series capillary high-performance liquid chromatography (HPLC).

Six microliters of digestion mixture was injected in to reverse phase (C_18_) nano online liquid chromatography coupled MS/MS analysis, using a GlycproSIL C_18_-80 Å (Glycpromass, Stove, Germany) with a length of 150 mm and a width of 0.075 mm. Mobile phase A consisted of water with 0.1% formic acid and mobile phase B consisted of acetonitrile with 0.1% formic acid. LC separation was carried out with a gradient from 10% to 95% and a flow rate of 0.2 µL/min.

The LC system was connected to a nanoelectrospray source of Thermo Scientific LTQ Orbitrap XL mass spectrometer (Thermo Fisher Scientific, Bremen, Germany) operated by Xcalibur 2.0.

The samples were analyzed with higher energy collisional dissociation, HCD, and collision induced dissociation, CID. For HCD fragmentation, Orbitrap survey scans were obtained in the mass range *m*/*z* 300–2000 and CID fragmentation performed with a target value of 5000 ions. When developing the method, samples were run in parallels of three, but as the method was established and the results found to be reproducible, this was reduced to two parallels for each sample.

The samples from one pneumococcal volunteer were run on a different Orbitrap, another Thermo Scientific LTQ XL mass spectrometer (Thermo Fisher Scientific, Bremen, Germany). LC separation was carried out on Dionex UltiMate 3000 RCLC nano system. Six microliters digestion mixture was injected in to reverse phase (C_18_) nano online liquid chromatography coupled MS/MS analysis with a length of 150 mm and a width of 0.075 mm, Acclaim PepMap C_18_-100 Å (Dionex, Thermo Scientific, Sunnyvale California, USA) with a trap column (Acclaim PepMap 100 C18, 5 µm, 100 Å, 300 µm i.d. 5 mm length with 10 µL/min flow rate).

### Mass spectrometry data analysis

Mass spectrometric data were analyzed using Thermo Scientific Xcalibur software version 2.0. The extracted ion chromatograms were presented for each glycoform with a mass tolerance of 5 ppm. The average of each peak height was calculated within the same retention time window and the MS/MS spectra were manually searched by Qual Browser version 2.0.7. The peaks were integrated and the values for each glycoform in charge state 2 and 3 were combined (see Table2 and Supplementary Table S2), and the values for all glycoforms added together. From this the percentage distribution of each glycoform was calculated. To compare different integration methods both the area of the peaks and the average height methods were tested, but as these gave the same results (data not shown), the two methods could be used interchangeably. The average height method was used in this report. Samples were made in duplicates and the variation measured was calculated. Statistical analysis was not carried out as the number of volunteers was too low to produce significant results.

## Results

The number of volunteers receiving each vaccine was limited and the results can therefore only provide indications of glycosylation trends, not statistical significance.

### Galactosylation

Galactose was presented as the total percentage of galactose-containing N-glycans.

#### The main responding IgG subclass

**Figure 4:**
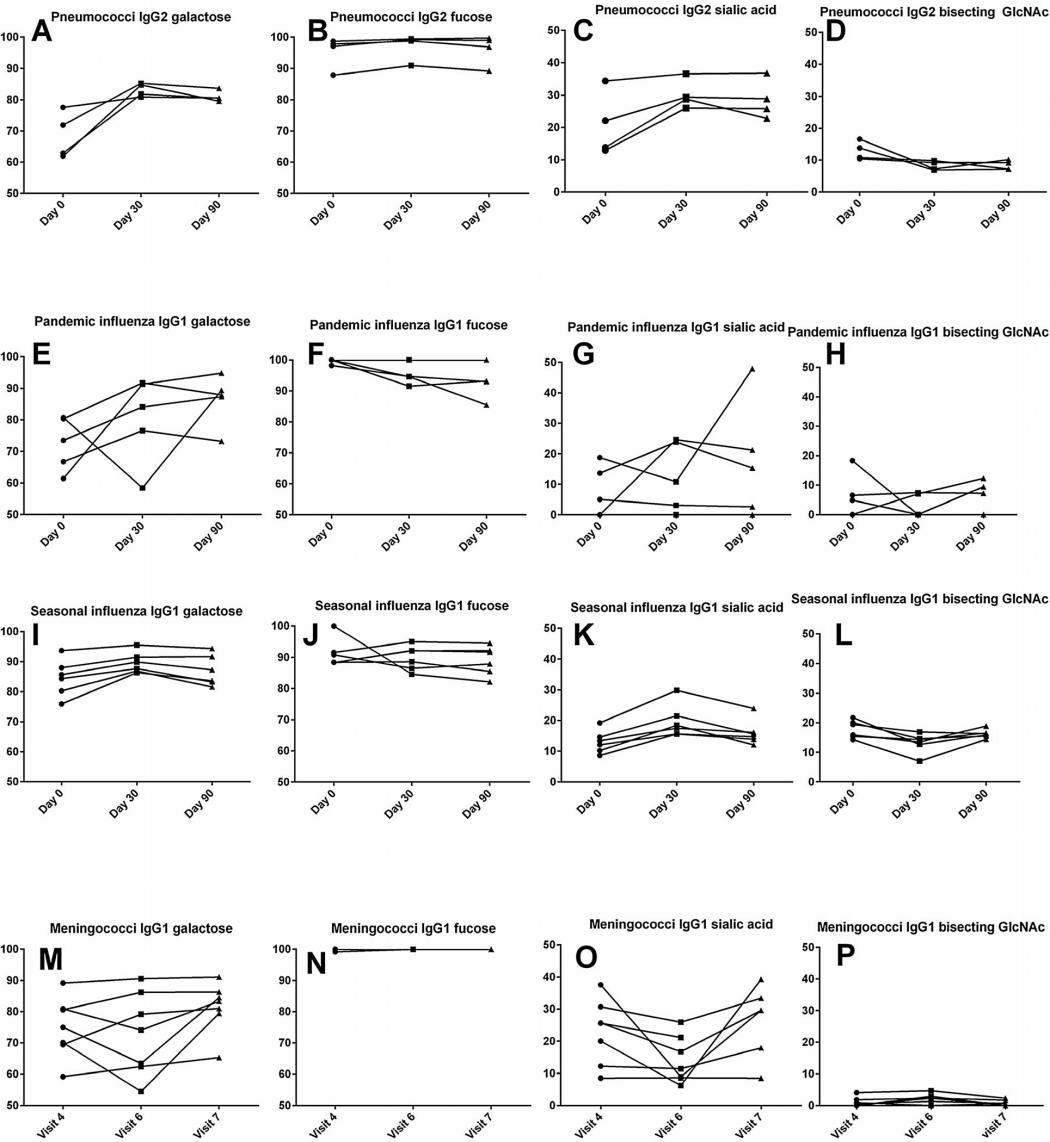
The three main time points for all four vaccines presented as percentage distribution of galactosylation, fucosylation, sialylation and bisecting GlcNAc. Pneumococcal IgG2 is displayed by A, B, C, and D while pandemic influenza IgG1 is presented as E, F, G, and H. I, J, K, and L show seasonal influenza IgG1 and M, N, O, and P show meningococcal IgG1. The time points for the meningococcal vaccine were visit 4 (1–2 weeks after third dose), visit 6 (10–12 months after third dose), and visit 7 (1–2 weeks after fourth dose). Two of the vaccinees that received the pneumococcal vaccine had their second and third sample collected at day 14 and day 26, not day 30 and day 90 as the others. The results were however similar to the others at day 30 and day 90, so to ease presentation their time points were presented as day 30 and 90, thus showing trend similarities. The *y*-axis portrays percentage distribution of glycoforms. See Table1 for full details of the meningococcal vaccine schedule.

All vaccines induced increased galactosylation from day 0 to day 30 after vaccination (see Fig. 4A, E, I, and M). From day 30 to day 90, galactose levels varied between different vaccines and for individual vaccinees; no change or a slight decrease for pneumococcal IgG2 while pandemic IgG1 either increased or decreased. For the extra pandemic influenza samples, an increase was seen at day 290 (vaccinee # 005) and day 559 (vaccinee # 006), both from the younger volunteers, while for one 65+ volunteer, a decrease was seen at day 201. Galactose levels in seasonal influenza IgG1 stayed stabile or decreased from day 30 to day 90, while meningococcal IgG1 increased after vaccination (visit 7) and showed inconsistent patterns between vaccinees after time.

#### The minor responding IgG subclass

Pneumococcal IgG1 and seasonal influenza IgG3 showed stable galactose levels (see Supplementary Table S3). Pandemic IgG3 showed an increase in galactose for most vaccinees at day 30, and a further increase or stable levels at day 90. The same trend regarding the late sampling days for IgG1 was seen for IgG3, an increase at day 290 (vaccinee # 005) and day 559 (vaccinee # 006) and a decrease at day 201 (vaccine # 009). Galactose levels for meningococcal IgG3 increased after vaccination, followed by a time dependent decrease.

### Fucosylation

#### The main responding IgG subclass

Fucose levels increased for the seasonal influenza from day 0 to day 30, and remained stable or decreased slightly for other vaccines. Overall, the fucose levels were close to or at 100% fucosylation (see Fig. 4B, F, J, and N).

#### The minor responding IgG subclass

Fucose levels of IgG1 from the pneumococcal vaccine stayed stable throughout (see Supplementary Table S3). Fucose levels in IgG3 for the other three vaccines differed between the vaccines, with seasonal influenza staying stable, pandemic influenza decreasing and meningococcal vaccine increasing. Pneumococcal IgG1 was completely fucosylated while seasonal influenza IgG3 had one vaccinee with stable levels, all the others were non-fucosylated. Pandemic IgG3 fucosylation decreased from day 0 to day 30 for almost all vaccinees, and decreased or stayed stable at day 90. Meningococcal IgG3 decreased at visit 6, followed by an increase at visit 7.

### Sialylation

#### The main responding IgG subclass

Sialic acid levels increased from day 0 to day 30 for all vaccines, and decreased for most vaccines from day 30 to day 90 (see Fig. 4C, G, K, and O). Pneumococcal IgG2 sialylation stayed stable from day 30 to day 90. Pandemic IgG1 sialylation was more varied, as amongst the 65+ volunteers there was either no sialic acid or a decrease at day 30, followed by an increase at day 90. The younger volunteers showed either low amounts with a steady decrease throughout or an increase at day 30 followed by a decrease at the next time points. The meningococcal vaccinees displayed increased IgG1 sialylation levels after each dose followed by a time dependent decrease.

#### The minor responding IgG subclass

Pneumococcal IgG1 sialylation stayed stable at 15–20%, while pandemic IgG3 had either no sialic acid or a continuous time dependent increase (see Supplementary Table S3). Seasonal IgG3 sialylation stayed stable and meningococcal IgG3 sialylation increased after each dose, followed by a time dependent decrease.

### Bisecting GlcNAc (*N*-acetylglucosamine)

#### The main responding IgG subclass

Pneumococcal IgG2 and both types of influenza IgG1 displayed a reduction from day 0 to day 30, followed by increased or stable levels. Meningococcal bisecting GlcNAc did not change much from one time point to the next (see Fig. 4D, H, L, and P).

#### The minor responding IgG subclass

The percentages of bisecting GlcNAC varied within each vaccine group (0–15%), but for each individual the levels stayed relatively stable or increased slightly (see Supplementary Table S3).

### Vaccine formulation affects glycosylation of the minor, but not the major responding subclass

The main responding IgG subclass displayed a similar glycosylation pattern, irrespective of whether it was IgG2 (pneumococcal) or IgG1 (the other three vaccines). The pneumococcal IgG1 response differed significantly from the IgG1 response from the other vaccines, as there were only minor changes in the pneumococcal IgG1 glycosylation pattern with time (see Supplementary Table S3).

### Squalene and glycoform complexity

**Figure 5:**
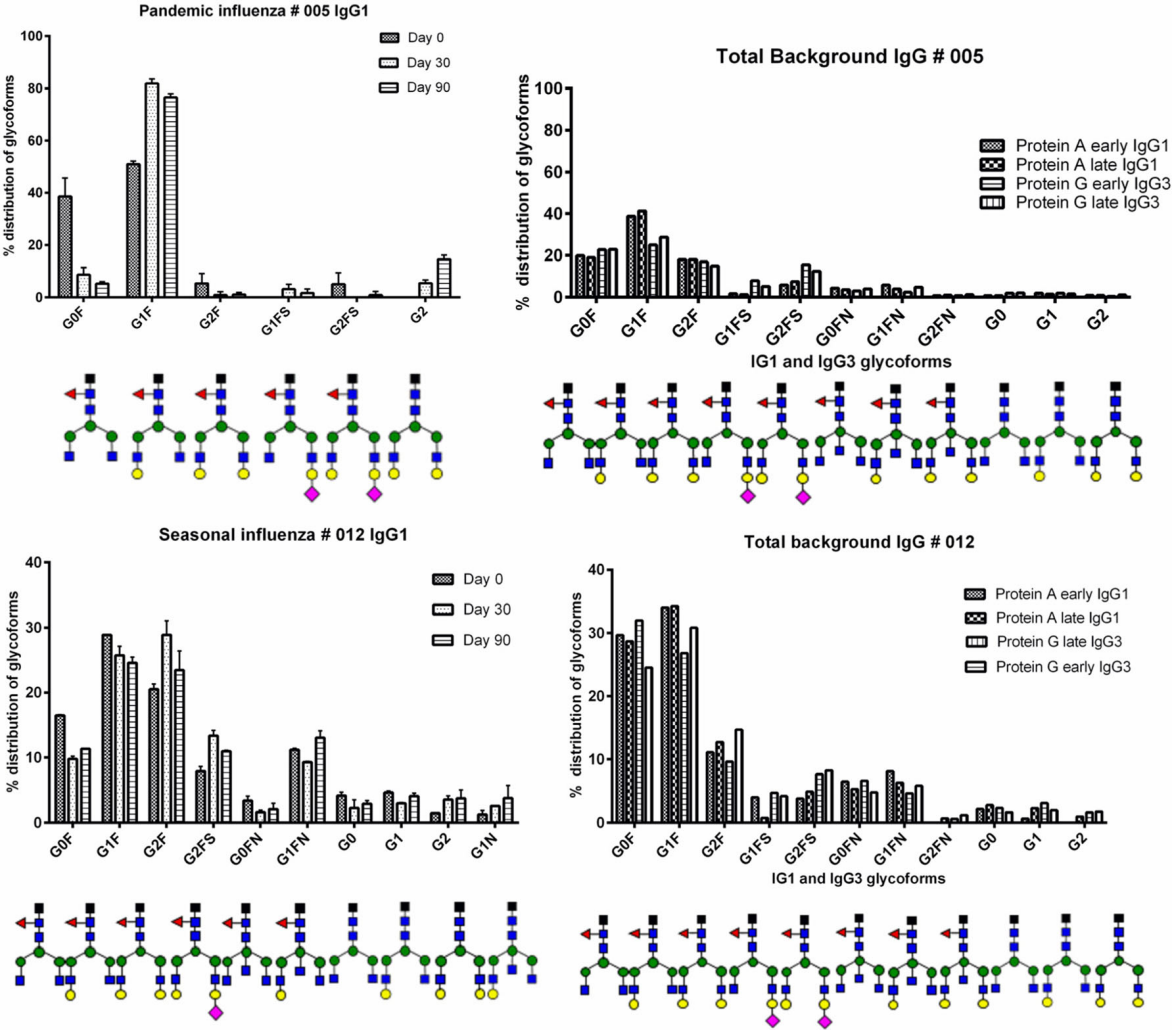
Glyform distribution of specific IgG1 (three time points) and total background IgG (two time points) for one vaccinee receiving the pandemic influenza vaccine and one vaccinee receiving the seasonal influenza vaccine. Glycoforms present at less than 1% were not included.

Two different types of influenza vaccine were analyzed, a seasonal influenza vaccine without an adjuvant and a pandemic influenza vaccine with the adjuvant squalene. The number of vaccinees was limited, but our results indicated that the pandemic influenza vaccine induced fewer different glycoforms (4–10 vs. 11–13) and these were of a less complex type, compared to the adjuvant-free seasonal influenza vaccine (see Fig. 5). The Al(OH)_3_ adjuvant in the meningococcal vaccine did not seem to cause this effect.

### Repeated annual vaccination did not alter the glycosylation pattern between seasons

**Figure 6:**
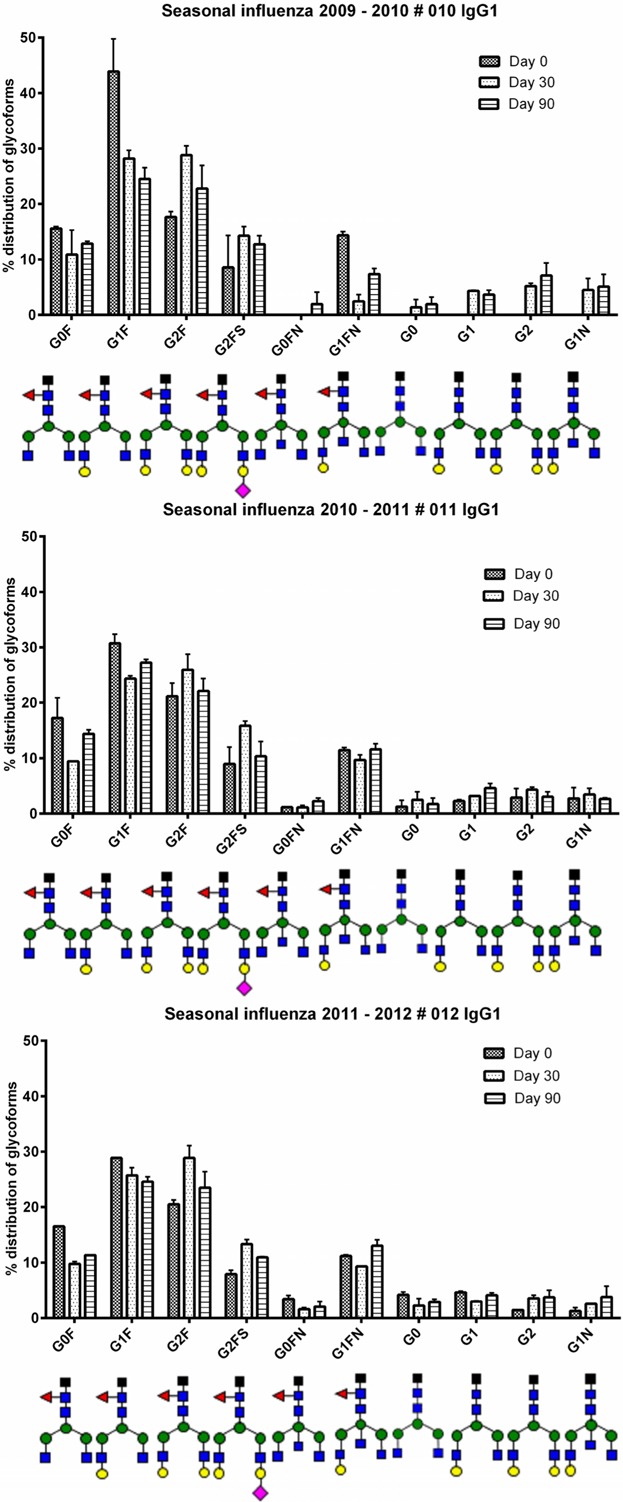
Glycoform distribution from one volunteer receiving the seasonal influenza vaccine for 3 consecutive years. Glycoforms present at less than 1% were not included.

For the seasonal influenza, three volunteers participated, but two volunteers received the vaccine for 2 or 3 consecutive years. Hence season 2009–2010 (vaccinee # 010), 2010–2011 (vaccinee # 011), and season 2011–2012 (vaccinee # 012) is all the same individual. Season 2010–2011 (vaccinee # 013) and 2011–2012 (vaccinee # 014) is the same individual. For the last volunteer, only one season was analyzed, 2011–2012, from vaccinee # 015. We found that being given the same vaccination for consecutive years resulted in proximately the same glycosylation pattern (see Fig. 4I, J, K, and L and Fig. 6).

### Differences in glycosylation between individuals

**Figure 7:**
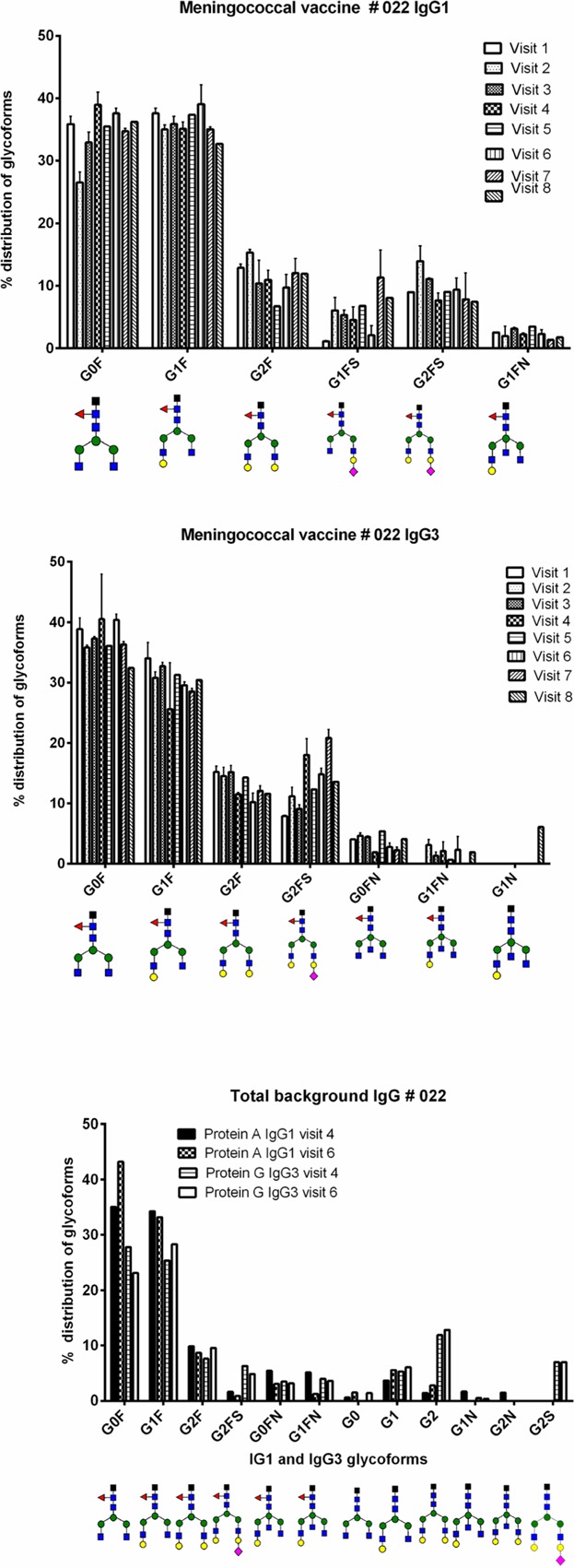
Glycoform distributions of IgG1, IgG3 (both eight time points), and total background IgG (two time points for one vaccine after four doses of the meningococcal vaccine. Glycoforms present at less than 1% were not included. See Table1 for full details of vaccine schedule.

Variation in glycoforms from one individual to another occurred. For meningococcal vaccinee # 022, eight time points were analyzed and the glycosylation patterns from one time point to the next were quite similar. Both IgG1 and IgG3 showed similar profiles to the other vaccines, but the IgG3 amino acid sequence differed. All the other vaccinees from all vaccine groups had an IgG3 with an amino acid sequence of EEQFNSTFR, while # 022 had EEQYNSTFR. This did however not change the IgG3 glycosylation profile drastically; though both specific and total background IgG3 showed a higher percentage of non-fucosylation compared to the other vaccines (see Fig. 7).

For the seasonal influenza vaccine, the two volunteers that received the vaccine for 2 or 3 consecutive years produced IgG3 glycoforms without sialic acid and fucose every year, the IgG3 glycoforms only contained galactose. The final vaccinee only received the vaccine 1 year, and produced IgG3 galactosylated glycoforms where 90% were fucosylated, but no sialic acid was observed (see Supplementary Table S3). Based on our limited number of volunteers, a difference between IgG1 and IgG3 fucosylation seemed to exist between individuals in this group.

### Glycosylation of background IgG

Total background IgG did not change significantly from an early time point to a later time point after immunization and negligible differences were seen between IgG1 and IgG3. Changes seen in the glycosylation pattern for specific IgG were therefore likely to be caused by the immunization. When combining total background IgG for all four vaccines, total background IgG1 eluted from protein A represented 54–86% galactose, 82–99% fucose, and 1–16% sialic acid. IgG3 eluted from protein G displayed 56–99% galactose (excluding pneumococcal vaccinees that had 39–76%), 73–97% fucose, and 0–31% sialic acid. Total background IgG2 from the pneumococcal vaccinees were eluted for protein A; displaying 46–73% galactose, 67–96% fucose, and 4–8% sialic acid. Background galactose levels for the two 65+ pandemic influenza vaccinees were 55–58% for IgG1 and IgG3, which was distinctly lower than the corresponding values in the younger volunteers; 69–75%.

## Discussion

The structure of the carbohydrates attached to asparagine 297 in the Fc portion of an IgG molecule has direct influence on effector functions and immune protective capacity. The aim of this pilot study was to analyze the antibody response after immunization with four different types of vaccines to determine glycosylation patterns, as this could prove beneficial in vaccine research and upon determining vaccine schedules. We have focused on Fc glycosylation which directly influence effector functions and thus the protective capacity of IgG molecules formed after vaccination. Fab can also be glycosylated in some circumstances; depending on the pathological state of the individual [31], but has no direct influence on effector function and was thus not relevant for our study. The vaccines were chosen in order to show influence on IgG glycosylation by varying the vaccine components. Furthermore, we wanted to investigate variation in IgG glycosylation over time since experiments in mice have indicated that especially sialic acid is increasing very late in the immune response, compatible with the notion that IgG acts anti-inflammatory when the acute risk phase during infection has passed [32]. It has been suggested that an IgG immune response has two phases, one pro-inflammatory shown in the acute phase during infection and possibly also right after a primary vaccination and one anti-inflammatory response during steady state, expected to be at later time points following immunization. A hallmark of such pro-inflammatory IgG activity could be lack of core-fucose, favoring ADCC activity and phagocytosis [4,33,34]. However, this hypothesis is largely based on experiments in mice and not in humans [16,17]. Thus results from human immunizations are much needed to clarify if a two-phase biological activity could be observed in vaccination situations in humans. Our present report is a pilot study providing indications of trends. However, we observed striking features which warrant further larger studies.

Based on our limited number of study subjects, we did not observe a prominent lack of IgG core-fucose shortly after immunization for any vaccinee compared to later time points. We also did not observe the hallmark for anti-inflammatory IgG; a noticeable increase in terminal sialic acid late in the response for any vaccine used. IgG1 responses after influenza and tetanus vaccination have recently been reported, displaying the same lack of non-fucose shortly after immunization and lack of increased sialic acid with time, but instead showed increased galactose and sialic acid shortly after vaccination. Serum was collected 3–5 weeks after each pandemic influenza vaccine dose in that study and showed steady sialic acid levels with time [18]. In the present report we extended this to IgG2 and IgG3 responses and included additional vaccines. We saw the same increase in sialic acid shortly after vaccination, but values varied at later time points. We do not necessarily dispute the possible presence of a two-phase IgG response under certain circumstances; a pro-inflammatory and an anti-inflammatory, but the basis for inducing and balancing these pronounced opposite IgGs seems elusive at least for humans. The dependence of these IgG variants upon the glycosylation patterns might also be more complex than previously suggested. The anti-inflammatory activity of sialic acid-rich human IgG seems well documented, although controversial. A resent paper showed that immune complexes with galactose-rich mouse IgG1 asserted anti-inflammatory properties by supporting the association of FcγRIIB with dectin-1 [8,35]. This complexity has been shown in mice infected with the yeast *Cryptococcus neoformans*, as administration of monoclonal IgG1 lead to the formation of a *C. neoformans*, glucuronoxylomannan and IgG complex, resulting in an acute lethal toxicity not seen with IgG3 [36]. When mice received IgG1 prior to *C. neoformans* infection, pro-inflammatory responses were diminished and granulocyte production increased to clear the infection [37]. Insufficient amount of antibodies may not cause the desired response, but excessive amounts may be counterproductive, as studies on *C. neoformans* showed interference with oxidative killing when antibody concentrations were markedly high [38]. The same antibody may exhibit pro- and anti-inflammatory properties depending upon the state of infection, type of microbe and antibody amount. All these factors contribute to a more complex picture than just glycosylation being the foundation of the two-phase IgG [39].

During a secondary antigenic exposure, memory B-cells and pre-existing Ag-specific antibodies are already present, and immune complexes may be formed with the exposed antigen, which may lead to higher antibody titers than caused by the antigen on its own [40,41]. An increase in harvest time for IgG antibodies resulted in less complex carbohydrate structures, possibly due to the enzymatic activity being put under strenuous pressure, resulting in incomplete carbohydrate structures [42,43]. Whether galactosylated antibodies are faster or easier to produce than non-galactosylated in vivo, or the formation of immune complexes with already existing antibodies leads to an increase in galactosylation, remains unknown.

An interesting observation was made following seasonal influenza vaccination as the IgG1 immune response exhibited mainly fucosylated glycoforms, while IgG3 glycoforms were mainly non-fucosylated. Total background IgG did not portray this pattern and it was therefore likely to be caused by the immunization, but was seen for this vaccine only. The number of volunteers receiving this vaccine was limited, but these preliminary results were seen for 2 or 3 consecutive years. Whether this was due to individual differences or the actual vaccine formulation is uncertain, but the difference was not observed when the same volunteers received other vaccines. We have previously studied recombinant chimeric mouse-human IgG3, which we found to contain more non-fucosylated glycoforms compared to IgG1 [42]. Interestingly, IgG3 antibodies in general appear superior to IgG1 regarding capacity for both ADCC and opsonophagocytic activity [11,44]. As ADCC is mainly activated through FcγRIIIa and removal of fucose caused a 50-fold increase in receptor-binding, the non-fucosylated IgG3 induced after seasonal influenza vaccination are likely to possess a strong capability for ADCC [10]. A difference in the hinge region between IgG1 (15 amino acids) and IgG3 (62 amino acids) could be one explanation for the glycoform differences [45,46], but no significant glycosylation differences were observed between recombinant wild type IgG3 and a hinge truncate mutant of this IgG3 [42,47].

Adjuvants are used to enhance the specific immune response [48–51]. Less complex glycoforms were observed by the low dose pandemic influenza vaccine containing the adjuvant squalene compared to the high dose, non-adjuvanted seasonal influenza vaccine, but importantly without the reduced fucose and sialic acid levels observed in the seasonal influenza IgG3 (see Fig. 5). This tendency might be caused by the low vaccine dose or the strong stimulant squalene having a direct influence on IgG glycosylation. The seasonal influenza vaccine also caused more diverse glycoforms, and the strong squalene-induced immune-stimulation could influence the kinetics of the immune response, resulting in a less matured IgG glycosylation. N-glycosylation has been improved in Congenital disorders of glycosylation upon inhibition of human squalene production [52,53]. Interestingly, the less potent meningococcal vaccine adjuvant did not seem to affect glycoform maturation. In a previous vaccine study with pandemic influenza vaccines, the adjuvant squalene emulsion MF59 did not cause such a difference [18,54]. Alterations in glycosylation were observed in rats receiving Freund's complete adjuvant to induce arthritis [55]. Mice immunized with 1–3 doses of ovalbumin showed increased levels of fucose upon repeated immunizations, while mice immunized with bovine serum albumin in incomplete Freund's adjuvant led to less galactosylated IgG antibodies as the anti-BSA titers increased [16,17]. Adjuvants may therefore by their own nature influence the antibody response and glycosylation. So far, we have not seen published reports on the consequences on antibody glycosylation caused directly by adjuvants in human vaccines.

Glycosyltransferases are enzymes that catalyze the transfer of a sugar moiety from donor sugars to acceptors, mainly in the Golgi apparatus for eukaryotic cells [56]. Changes or stimulations to the glycan-producing cell may affect enzyme concentration, which is likely to influence glycosylation, for example the altered distribution of the serum glycoprotein TRF in chronic alcoholics compared to healthy individuals [57]. Treating B-cells with factors that stimulate the innate immune system or cytokines from the adaptive immune system may modulate IgG Fc-glycosylation, such as Interleukin 21 altering the factors bringing glycosyltransferases and IgG1 together in the Golgi [58]. In our study, we saw considerable variations in glycoforms from one individual to another, indicating that glycosylation is a flexible process harboring several parameters which varies among individuals. IgG glycosylation also differs between species, as seen with species-specific activity of β-1,4-galactosyltransferase [13]. Analysis of IgG Fc-glycosylation of 2300 individuals revealed that pure competition between glycosyltransferases in vivo was not significant, suggesting a more regulated biological process [59]. Glycosyltransferases constitute a vital part of this process. Correlations between effector function and glycosyltransferase expression were found in glycosyltransferase gene expression in B cells in HIV patients, producing HIV-1-specific antibodies glycosylated in a manner to enhance ADCC activity. Such a differentiation could be used in vaccine formulations to enhance antibody activity against pathogens [60].

The IgG subclass dominating the response induced by a polysaccharide vaccine is IgG2 [20,61]. The IgG2 glycosylation pattern from the pneumococcal vaccine did not differ significantly compared to the main IgG1 immune response from the other vaccines. The antibody response toward polysaccharides, as opposed to proteins, is generally T-cell independent, and the responding B-cells thus need less T-cell help in their development toward antibody producing plasma cells. Our pilot study indicates that such T-cell help toward B-cells has minor influence on the glycosylation pattern of downstream plasma cell production of IgG. Furthermore, the minor IgG1 subclass response from the pneumococcal vaccine differed compared to the IgG1 response from the others (see Fig. 4 and Supplementary Table S3).

Quantifiable IgG titers have been measured 2–4 weeks after immunization with pneumococcal polysaccharide vaccines, seasonal influenza vaccines, and MenBVac® [19,29,62–65], and differences in immune response time between antigens were therefore not likely to cause the variety in IgG glycosylation.

Antibodies kill or neutralize pathogens by phagocytosis and serum bactericidal activity (SBA) [66,67], and antibody function is affected by glycosylation [10,14,58,68]. Serum bactericidal activity (SBA) and opsonic activity have previously been published for the vaccinees receiving the meningococcal vaccine, measuring activity by reciprocal antibody titers [69]. No glycosylation pattern trends were seen for SBA, but three vaccinees displayed changes in opsonic activity that could be related to glycosylation [69]. Vaccinees # 017 and # 020 showed a large increase in opsonic activity from visit 6 to visit 7 (see Supplementary Table S4) with a parallel increase in IgG1 and IgG3 galactosylation and sialylation, and increased fucosylation for IgG3 antibodies. Vaccinee #018 showed a slight increase in opsonophagocytic activity (see Supplementary Table S4) with a concurrent IgG1 and IgG3 sialylation increase. While the specific antibody concentration did not change significantly between these two visits for vaccinee #018, a clear increase was seen for both IgG1 and IgG3 in vaccinees #017 and #020 (see Supplementary Table S1).

In conclusion, we have analyzed serum or plasma from volunteers after immunization with four different vaccines and determined that the majority of vaccinees showed distinct increases in IgG galactosylation and sialylation following immunization. Changes in IgG fucosylation following immunization seemed vaccine dependent and several vaccines resulted in fully fucosylated IgG, while non-fucosylated IgG3 was seen for some individuals after seasonal influenza immunization. Differences were seen in complexity and variability of glycoforms between influenza vaccines with and without adjuvant. The number of volunteers in this pilot study was limited, but glycosylation patterns seemed to be affected by both type and intervals of immunization. Combining larger studies with biological data could result in findings proving advantageous for optimizing vaccine formulation and vaccination schedules.

## SUPPORTING INFORMATION

**Table S1.** Specific IgG1 and IgG3 antibodies from meningococcal vaccinees, portrayed as µg/mL. **Table S2**. Displaying mass to charge (*m*/*z*) of 20 IgG3 glycoforms with a different amino acid sequence. **Table S3**. The minor responding subclass from the different vaccines, displayed as increase (↑), decrease (↓), or no change (↔) in sugar composition observed at day 30 and day 90 after immunization, compared to the sample taken before immunization. Two of the vaccinees that received the pneumococcal vaccine had their second and third sample collected at day 14 and day 26, not day 30 and day 90 as the others. The results were however similar, so to ease presentation their time points were presented as day 30 and 90. **Table S4**. The seven meningococcal vaccinees were previously tested for serum bactericidal activity and opsonaphagocytic activity. Results are shown as reciprocal titers, published previously [60].
