# Supplementary material for: A pilot study showing differences in glycosylation patterns of IgG subclasses induced by pneumococcal, meningococcal, and two types of influenza vaccines
Source: Immun Inflamm Dis. 2014 May 22;2(2):76–91. doi: 10.1002/iid3.22 (PMC4217548; doi:10.1002/iid3.22)
Supplement: Supplementary file 1 [file iid30002-0076-SD1.docx]

**Supplementary material**

| **Supplementary Table 1: Specific IgG1 and IgG3 antibodies from meningococcal vaccinees, portrayed as μg/ml.** | | | | | | |
| --- | --- | --- | --- | --- | --- | --- |
|  | **IgG1 visit 4** | **IgG1 visit 6** | **IgG1 visit 7** | **IgG3 visit 4** | **IgG3 visit 6** | **IgG3 visit 7** |
| **# 016** | 15 | 7.5 | 6.6 | 2.3 | 0 | 2.0 |
| **# 017** | 11.3 | 0.9 | 3.8 | 3.4 | 0 | 5.2 |
| **# 018** | 26.3 | 5.6 | 6.3 | 5.2 | 0 | 0.9 |
| **# 019** | 2.6 | 1.3 | 1.6 | 0.8 | 0.2 | 0.4 |
| **# 020** | 21.3 | 5.6 | 9.8 | 7.8 | 0 | 4.7 |
| **# 021** | 22.5 | 5.6 | 7.0 | 3.8 | 0 | 7.5 |
| **# 022** | 15 | 5.6 | 6.5 | 1.1 | 0 | 5.6 |

| Supplementary table 2: Displaying mass to charge (*m/z*) of 20 IgG3 glycoforms with a different amino acid sequence | | |
| --- | --- | --- |
|  | IgG3 | |
|  | Charge state 2 | Charge state 3 |
|  | IgG3 EEQYNSTFR | |
| G0F | **1309.5291** | **873.3551** |
| G1F | **1390.5555** | **927.3727** |
| G2F | **1471.5819** | **981.3903** |
| G1FS | **1536.1032** | **1024.4045** |
| G2FS | **1617.1296** | **1078.4221** |
| G0FN | **1411.0688** | **941.0482** |
| G1FN | **1492.0952** | **995.0659** |
| G2FN | **1573.1216** | **1049.0835** |
| G1FNS | **1637.6429** | **1092.0977** |
| G2FNS | **1718.6693** | **1146.1153** |
| G0 | **1236.5002** | **824.3334** |
| G1 | **1317.5266** | **878.6868** |
| G2 | **1398.5530** | **932.7044** |
| G1N | **1419.0663** | **946.3799** |
| G0N | **1338.0400** | **892.3623** |
| G2N | **1500.0926** | **1000.3975** |
| G1S | **1463.0742** | **975.7186** |
| G2S | **1544.1007** | **1029.7362** |
| G1NS | **1564.6140** | **1043.4117** |
| G2NS | **1645.6400** | **1097.4294** |

| **Supplementary table 3: The minor responding subclass from the different vaccines, displayed as increase (↑), decrease (↓) or no change (↔) in sugar composition observed at day 30 and day 90 after immunization, compared to the sample taken before immunization.** Two of the vaccinees that received the pneumococcal vaccine had their second and third sample collected at day 14 and day 26, not day 30 and day 90 as the others. The results were however similar, so to ease presentation their time points were presented as day 30 and 90. | | | | | | | | |
| --- | --- | --- | --- | --- | --- | --- | --- | --- |
|  | **GALACTOSE** | | **FUCOSE** | | **SIALIC ACID** | | **BISECTING GlcNAc** | |
|  | **Day 30** | **Day 90** | **Day 30** | **Day 90** | **Day 30** | **Day 90** | **Day 30** | **Day 90** |
| **Pneumo-coccal vaccine (IgG1)** | ↔ | ↔ | ↔ | ↔ | ↔ | ↔ | ↑ | ↔ |
| **Pandemic influenza vaccine (IgG3)** | ↑ | ↓↔ | ↓ | ↑↓ | ↑↔ | ↔ | ↔ | ↔ |
| **Seasonal influenza vaccine (IgG3)** | ↔ | ↔ | ↔ | ↔ | ↔ | ↔ | ↔ | ↔ |
|  | **10-12 months after 3^rd^ dose** | **1-2 weeks after 4^th^ dose** | **10-12 months after 3^rd^ dose** | **1-2 weeks after 4^th^ dose** | **10-12 months after 3^rd^ dose** | **1-2 weeks after 4^th^ dose** | **10-12 months after 3^rd^ dose** | **1-2 weeks after 4^th^ dose** |
| **Meningo-coccal vaccine (IgG3)** | ↓ | ↑ | ↓ | ↑ | ↓ | ↑ | ↑ | ↓↔ |

| **Supplementary table 4: The 7 meningococcal vaccinees were previously tested for serum bactericidal activity and opsonaphagocytic activity. Results are shown as reciprocal titres, published previously [60].** | | | | |
| --- | --- | --- | --- | --- |
|  | **Serum bactericidal activity** | | **Opsonophagocytic activity** | |
|  | **Visit 6** | **Visit 7** | **Visit 6** | **Visit 7** |
| **Vaccinee # 016** | 128 | 128 | 128 | 128 |
| **Vaccinee # 017** | ˂2 | 8 | 8 | 64 |
| **Vaccinee # 018** | 8 | 8 | 32 | 64 |
| **Vaccinee # 019** | ˂2 | ˂2 | ˂2 | 8 |
| **Vaccinee # 020** | 2 | 4 | 32 | 64 |
| **Vaccinee # 021** | 8 | 2 | 16 | 16 |
| **Vaccinee # 022** | 16 | 16 | 16 | 32 |
